# Prevalence and risk factors of sexually transmitted infections among French service members

**DOI:** 10.1371/journal.pone.0195158

**Published:** 2018-04-02

**Authors:** Sandrine Duron, Henri Panjo, Aline Bohet, Christine Bigaillon, Sébastien Sicard, Nathalie Bajos, Jean-Baptiste Meynard, Audrey Mérens, Caroline Moreau

**Affiliations:** 1 French Military Center for Epidemiology and Public Health, Marseille, France; 2 Aix Marseille Univ, INSERM, IRD, SESSTIM, Sciences Economiques & Sociales de la Santé & Traitement de l’Information Médicale, Marseille, France; 3 Gender, sexual and reproductive health, Centre for research in Epidemiology and Population Health, U1018, Inserm, Villejuif, France; 4 Medical biology laboratory, French military hospital Bégin, Saint-Mandé, France; 5 French Military Medical Academy, Ecole du Val-de-Grâce, Paris, France; 6 Department of Population, Family and Reproductive Health, Johns Hopkins Bloomberg School of Public Health, Baltimore, Maryland, United States of America; University of Westminster, UNITED KINGDOM

## Abstract

**Introduction:**

Sexually Transmitted Infections (STIs) have always represented a public health concern in the military, yet most studies rely on self-reports among non-random samples of military populations. In addition, most of the studies exploring STI rates among the military focus on US service members. This paper assesses the prevalence and correlates of STIs in the French military using biomarkers and compares self-reported versus diagnosed STIs.

**Methods:**

Data are drawn from the COSEMIL study, a national sexual health survey conducted in the French military in 2014 and 2015. A random sample of 784 men and 141 women aged 18–57 years completed a self-administered questionnaire and provided biological samples for STI testing. We used logistic regression modeling to identify the correlates of STI diagnosis and self-reports.

**Results:**

The prevalence of diagnosed STIs was 4.7% [3.8–5.9], mostly due to *Chlamydia trachomatis*. This rate was four times higher than the 12 months self-reported rate of 1.1% [0.6–2.3]. Reported STI rates were similar among men and women (1.1% *versus* 1.8%), but diagnosed STI rates were twice as high among females *versus* males (10.4% *versus* 4.1%, p = 0.007). There were significant differences in the determinants of reported *versus* diagnosed STIs. In particular, age and sexual orientation were associated with reported STIs, but not with diagnosed STIs. Conversely, STI counseling and depression were associated with STI diagnosis but not with STI reports.

**Conclusion:**

This study underlines the need to use biomarkers in population-based surveys, given the differential and substantial underreporting of STIs. Results also highlight the need for programmatic adaptation to address gender inequalities in STI rates, by developing women’s health services in the French military. Addressing such needs not only benefits women but could also serve as a strategy to reduce overall STI rates as most military women have military partners, increasing the risk of internal transmission.

## Introduction

Sexually Transmitted Infections (STIs) have always represented a public health concern in the military, from pre-antibiotic era during World war I to more contemporary threats related to HIV in the 1980s [1–3] and drug resistant Gonorrhea infections in recent years [4–6]. The most common bacterial STIs (*Chlamydia trachomatis* and *Neisseria gonorrheae*) are curable, yet untreated, these infections cause substantial sequelae, increasing the risk of pelvic inflammatory disease, infertility and ectopic pregnancy [4,7–9]. Military recruits have traditionally been considered a high-risk group for STIs due to their younger age, lower socio-economic status and high mobility [10]. In addition, while expressions of masculinities vary across and within military settings, hegemonic forms of masculinities combined with a culture of risk [11–15] are conducive of sexual risks taking, including multiple partnerships and non-condom use [11,12,16]. Studies of sexual behaviors in the military consistently report high levels of transactional sex, concurrent partnerships, and low levels of condom usage, all of which contribute to STI acquisition [11,17–20]. A number of recent studies also indicate high rates of binge drinking among military personnel, reported to heighten sexual risk taking [17,18,21–24]. Finally, STI rates are also conditioned on professional transience, with potential heightened risk of transmission during deployment in high prevalence regions [4,25].

While many studies, mostly conducted in the US, report higher incidence of STIs in the military compared to the general population [10,26,27] not all studies concur [28], raising attention to the selection of study populations (mostly based on convenience samples or clinic based samples) and STI ascertainment. Most research on STIs in the military is based on retrospective surveillance registry data that include a large number of certified cases. While such studies are useful in estimating levels and trends in STI rates as well as in identifying socio-economic and military conditions related to STI portage, their interpretability is questionable due to gross differential under-reporting (up to 30% of underreporting compared to direct ascertainment using biomarkers [29–31]) non-detection of asymptomatic cases and non-reporting of STIs treated outside of the military healthcare system.

Military institutions have generally responded by offering comprehensive STI prevention, including sexual health education, screening, post exposure prophylaxis and epidemiological surveillance [25,32]. The French military has implemented such a program, with reinforcing messages before deployment. The epidemiological surveillance system estimates a prevalence rate of 42 p. 100 000 servicemen for the period 2014–2016 (unpublished data), but these estimates are likely under-estimated due to non-systematic screening. Moreover, the system fails to identify the circumstances leading to STI acquisition limiting its ability to inform the changing ecology of STIs in the French armed forces, typified by growing feminization and professionalization.

To address this gap, the COSEMIL survey was designed to explore current sexual health issues among French armed forces, including STI infections. This paper has two objectives: 1) estimate the prevalence and correlates of STI infections among servicemen and servicewomen based on biomarkers, 2) compare results using self-reported versus diagnostic STIs (based on biomarkers) to assess potential bias from self-reported STI measures used in most population based surveys [33].

## Methods

### Study procedures

Data are drawn from the COSEMIL study (COMportement SExuel des MILitaires), a national survey on sexual health in the French military conducted in 2014 and 2015. The overall objective of the COSEMIL study was to explore sexual norms and practices among a representative sample of the French armed forces and their relation to multiple dimensions of sexual health. A two-stage probability sampling strategy was used to identify 12 military units stratified by geography and military branch, followed by the random selection of 120 service members per unit. Individuals were eligible if they were 18 years or more and women were oversampled (1 woman for 5 men). Upon selection, participants attended an information session and at the end of the session were asked to give two written consent if they agreed to participate. The first consent was for a self-administered survey, the second to undergo biological testing for STIs. Participants could select out of the biological tests while still completing the questionnaire. A single anonymous identifier permitted a linkage between survey responses and biological tests. The correspondence between the respondent’s contact information and the anonymous identifier was kept in locked cabinet under the responsibility of the main investigator and destroyed after results were sent out to each respondent. The COSEMIL survey received the approval of the French government ethical oversight agency (Commission Nationale Informatique et Liberté, N° 2014–100).

**Fig 1:**
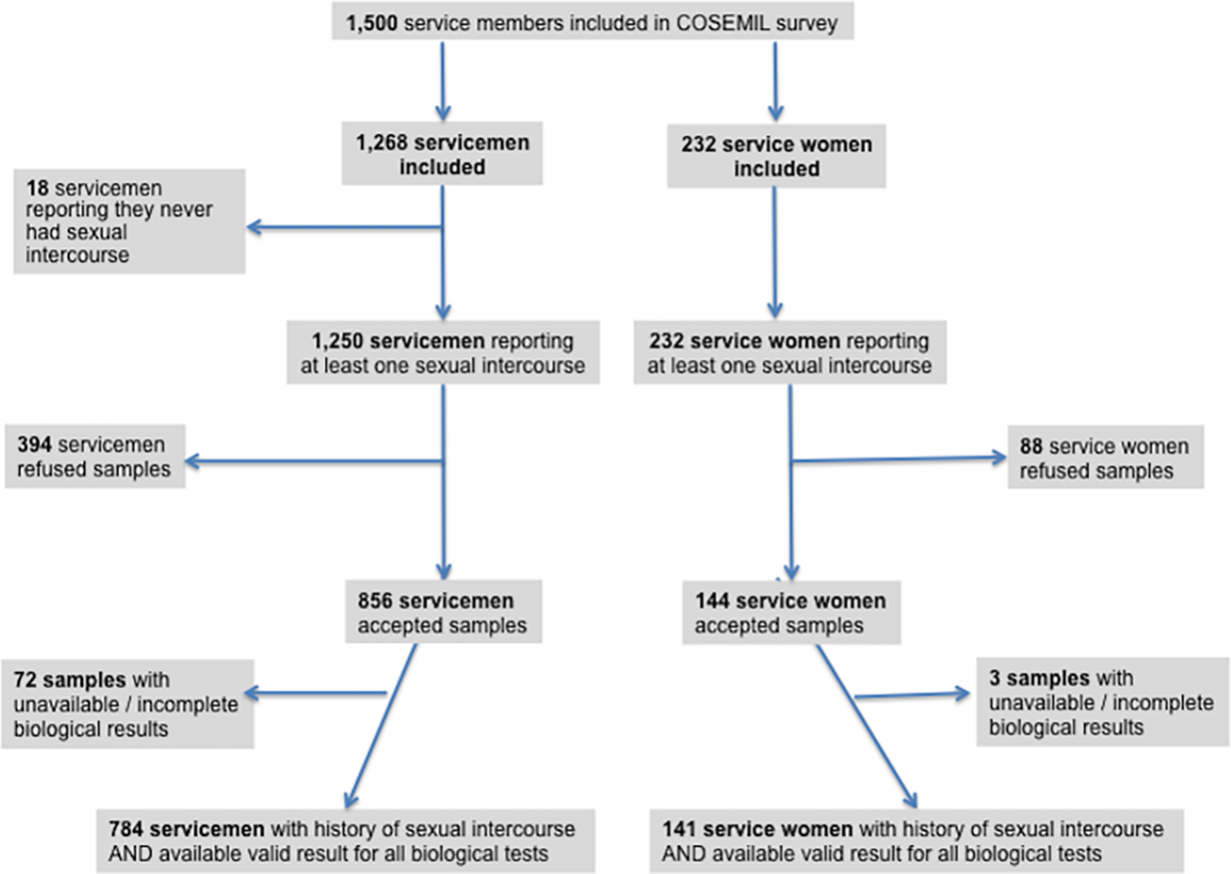
Flow chart for the analyses on lower STIs, COSEMIL survey.

A total of 1,692 military personnel attended the information session and 1,500 provided written consent to participate in the survey (178 individuals declined participation and 14 questionnaires were lost due to software deficiency) (Fig 1). For this analysis, we excluded 18 participants who had never had sexual intercourse, and 482 individuals who declined biological tests. In addition, we excluded 75 participants whose biological tests were incomplete or not available (insufficient quantity, strong hemolysis, PCR inhibitors). Our final study population comprised 784 men and 141 women aged 18–57 years.

Participants first completed a self-administered questionnaire on laptop computers collecting information on their socio-demographic background and a range of topics related to their sexual lifestyles and sexual health. The study also examined other health related topics including mental health issues and general perceived health. After completing the questionnaire, respondents who provided consent, were tested for the following bacterial infections (*Chlamydia trachomatis*, *Neisseria gonorrhoeae* and *Mycoplasma genitalium*), and systemic STIs. Specifically, they provided a blood sample (2 tubes) to screen for human immunodeficiency virus (HIV), hepatitis B (HBV) and C (HCV) and syphilis. Diagnoses were based on HIV ELISA serology combining Ac-antiHIV1/2 and AgP24 detection, HBV, HCV and syphilis serologies. All analyses were performed using tests that were certified for in vitro diagnosis (CE-IVD) in order to return results to each patient. Additionally, males were asked to provide a first void urine sample and females were asked to provide a self-collected vaginal swab (Sigma VCN, Elitech) to screen for the 3 aforementioned bacterial STIs, using a validated nucleic acid amplification test with real-time PCR method. Detailed diagnosis methods are available as supporting file (S1 Appendix). All biological specimens were collected on military settings and samples were transported and processed at the Begin Military Hospital laboratory. Results were sent out to each participant and if positive, a prescription for a repeat test was included. All additional medical tests and treatments were covered by the French military health insurance. Respondents could choose to consult military healthcare services or civilian health services at no cost for further tests and treatment in case of positive results.

### Measures

The present study focuses on STI diagnoses and on STI reporting defined as follows:

- STI diagnoses corresponded to biologically confirmed STIs based on the results of the blood, urine and vaginal tests. Urine or vaginal samples positive for any of the 3 pathogens (*C*. *trachomatis*, *N*. *gonorrhoeae* and *M*. *genitalium*) were considered as confirmed cases of lower genital tract infection. In addition, positive HIV or HBV (Ag HBs) serologies were considered as confirmed cases of systemic STIs (acknowledging that HBV is not only sexually transmitted). In the case of syphilis, patients with positive TPLA and VDRL tests were considered as confirmed cases, while patients with positive TPLA but negative VDRL were classified as negative cases because repeated tests were all negative.

- STIs reported in the last 12 months. This measure was assessed by asking respondents if they “ever had an STI” and the date of the last infection. In addition, respondents were asked to indicate the type of last infection among the following list “Syphilis/ Genital herpes/ *Chlamydia* / Condyloma/ HIV or AIDS/ Gonorrhea/ *Trichomonas*/ *Mycoplasma*/ *Papillomavirus*/ Other (to be specified)”. In the 12 months prior to the survey, respondents only reported Syphilis, *Chlamydia* and Gonorrhea infections.

We considered the following factors related to STI infections, including respondents’ socioeconomic background, sexual lifestyle, STI knowledge and counseling and mental health indicators. Sexual lifestyle information related to sexual orientation, lifetime experienced of commercial sex and lifetime experience of forced sex. We also considered current sexual and partnership indicators including partnership and cohabitation status, number of partners in the last 12 months, type of partner at last sex (regular or casual), type of sexual practices in the last 12 months (vaginal, anal and oral) and use of condom at last sexual intercourse. Two questions assessed respondents’ exposure to STI counseling and screening. Finally, we included a measure of depression evaluated using the CESD-10 scale [34]. We considered a score of 10 (out of 30) as the threshold for depressive symptoms [34,35]. Hazardous and harmful consumption of alcohol was measured using the AUDIT C score categorized in three groups (no consumption = 0 /non risk consumption (1 to<4 for women and 1 to <5 for men)/ and high risk consumption (≥ 4 for women and ≥ 5 for men) [36,37].

### Statistical analysis

After describing the study sample (n = 925) and assessing the potential differences with participants who did not have a biological result (n = 557), we followed a three-step approach. First, we estimated the prevalence of STI diagnosis and conducted bivariate and multivariate analysis using logistic regression modeling to identify the correlates of STI diagnosis. We selected factors known to be related to STIs based on previous studies as well as factors identified in bivariate analysis with a p-value below 0.20. Second, we conducted bivariate analysis of factors related to STI reporting in the last 12 months in order to identify differences in the determinants of reported STIs versus diagnosed STIs. Finally, to assess potential bias due to sample selection (38% of respondents did not have a biological test and therefore were not included in our analysis), we fitted a maximum pseudo-likelihood probit model with sample selection which jointly models (probit models) the probability of selection into the study (having a usable biological test), the probability of STI diagnosis among those with observed data and a correlation coefficient ρ between model residuals (unobservables that determine study participation and unobservables that determine STI diagnosis). If ρ is significantly different from 0, standard regression models on the basis of "only complete observations" yields biased and inconsistent estimates (STI diagnosis outcome). Maximum pseudo-likelihood probit model with sample selection corrects for this potential bias and also provides consistent and asymptotically efficient estimates for all parameters in the models [38].

All analyses were stratified by sex in order to account for sex-differences in determinants of sexual behaviors and outcomes. All analyses were weighted to take into account the complex sampling design of the COSEMIL study and post stratification was applied to replicate the age, sex, military branch and rank distribution of the French military workforce. Analyses were performed using Stata 14 software.

## Results

### Characteristics of the study population

The sociodemographic characteristics of the study sample (n = 925) are described in Table 1. Comparison between included and excluded respondents showed study participants were more likely to be educated and to have been born outside of Mainland France. Female participants were less likely to have a partner at the time of the survey (as compared to excluded females) while male participants were less likely to indicate they had no financial problem at the time of the survey (as compared to excluded males).

**Table 1:** Sociodemographic characteristics of the study sample (n = 925) and of respondents excluded from the analysis (n = 557), COSEMIL survey.

	Men			Women		
	Study sample^a^ % ^c^ (N)	Excluded sample^b^ % ^c^ (N)	p	Study sample^a^ % ^c^ (N)	Excluded sample^b^ % ^c^ (N)	p
**Total**	**63.5 (784)**	**36.5 (466)**		**54.6 (141)**	**45.4 (91)**	
**Age years**			0.534			0.036
<25	20.0 (154)	16.9 (78)		25.7 (40)	8.3 (15)	
25–30	27.1 (202)	29.0 (125)		39.7 (43)	44.1 (39)	
>30	52.9 (428)	54.1 (263)		34.6 (58)	47.6 (37)	
**Cohabitation status**			0.28			<0.001
Lives with a partner all of the time	57.2 (469)	59.9 (292)		44.5 (67)	76.0 (57)	
Lives with a partner part of the time	25.7 (184)	22.1 (94)		29.8 (43)	15.1 (21)	
No current partner	17.1 (130)	18.0 (80)		25.6 (30)	8.9 (13)	
**Children**						
No	45.7 (347)	43.2 (198)	0.47	71.3 (86)	52.0 (54)	0.05
Yes	54.3 (436)	56.8 (267)		28.7 (55)	48.0 (37)	
**Level of education**			0.019			0.03
<High school graduation	39.9 (313)	46.8 (218)		24.5 (36)	36.5 (33)	
High school graduation	35.0 (300)	34.0 (163)		52.8 (77)	38.1 (37)	
>High school	25.1 (169)	19.1 (85)		22.7 (27)	25.4 (21)	
**Financial situation**			0.001			0.202
No problem	40.4 (352)	49.0 (230)		52.6 (74)	43.2 (48)	
Tight or difficult	59.6 (430)	51.0 (234)		47.4 (66)	56.8 (43)	
**Born in mainland France**			0.02			0.022
No	15.0 (114)	10.3 (49)		15.1 (22)	6.9 (8)	
Yes	85.0 (670)	89.7 (417)		84.9 (119)	93.1 (83)	
**Importance of religion**			0.266			0.841
Important/very important	24.8 (149)	22.3 (83)		18.7 (33)	17.8 (16)	
Not very important/ not important	48.2 (409)	46.7 (217)		48.6 (67)	52.2 (44)	
No religion	27.1 (218)	31.0 (160)		32.7 (41)	30.0 (31)	
**Army rank**			0.464			0.064
Enlisted personnel	41.9 (336)	44.4 (213)		42.2 (67)	52.6 (52)	
Non commissioned officer	46.7 (373)	42.1 (211)		48.7 (65)	44.4 (34)	
Officer	11.4 (75)	13.5 (42)		9.0 (9)	3.1 (5)	
**Type of Army**			0.759			0.471
Army rank	59.9 (351)	64.6 (221)		41.2 (46)	37.6 (27)	
Air Force	18.4 (238)	18.5 (137)		38.1 (55)	50.2 (42)	
Navy	21.7 (195)	17.0 (108)		20.7 (40)	12.2 (22)	
**Number of years in the army**			0.903			0.111
1–10	43.8 (323)	43.6 (185)		66.8 (85)	47.2 (52)	
>10	56.2 (459)	56.4 (279)		33.2 (56)	52.8 (39)	

^a^ This study sample includes respondents who ever had sexual intercourse with an available biological testing.

^b^ The excluded sample includes respondents who ever had sexual intercourse with no available result of biological testing.

^**c**^ All percentages are weighted to account for the complex sampling design and post stratification.

Sexual and reproductive health indicators are presented in Table 2. Altogether 15% of women and 1.9% of men reported same sex relationships. The same percentage of women and men reported more than two sexual partners in the last 12 months (33.3% *versus* 29.6%, p = 0.31) but women were more likely to indicate not using a condom at last sex with a casual partner (10.8% *versus* 5.4%, p = 0.03). 41% of men indicated lifetime experience of commercial sex. Women were more likely to report lifetime experience of forced sex than men (7.4% *versus* 1.8%, p = 0.008). Women were also more likely to have ever been tested for STIs (64.2% *versus* 39.4%) while men were more likely to have received STI counseling (74.1% *versus* 55.8%). Comparisons between our study sample and respondents who were excluded indicate that a greater number of male study participants reported ever having had commercial sex, ever been screened for STIs as compared to those excluded. Male participants were also more likely to report multiple sex partners in the last 12 months. Likewise, female participants were more likely to report several sex partners in the last year and more likely to indicate non-use of condoms with a casual partner at last sex as compared to female non-participants.

**Table 2:** Sexual lifestyle and mental health indicators characteristics of the study sample (n = 925) and of respondents excluded from the analysis (n = 557), COSEMIL survey.

	Men			Women		
	Study sample ^a^ % ^c^ (N)	Excluded sample ^b^ % ^c^ (N)	p	Study sample ^a^ % ^c^ (N)	Excluded sample ^b^ % ^c^ (N)	p
**Sexual orientation**			0.628			0.586
Exclusively heterosexual	98.1 (770)	98.6 (463)		86.1 (123)	82.9 (77)	
Homo/Bisexual	1.9 (14)	1.4 (3)		13.9 (18)	17.1 (14)	
**Condom at first sex**			0.303			0.5
No	30.2 (250)	32.9 (132)		25.2 (33)	31.6 (19)	
Yes	69.8 (532)	67.1 (328)		74.8 (107)	68.4 (71)	
**Ever had commercial sex**			0.044			0.341
No	58.7 (459)	66.3 (308)		99.2 (140)	100.0 (91)	
Yes	41.3 (325)	33.7 (158)		0.8 (1)	0.0 (0)	
**Ever experienced forced sex**			0.573			0.059
No	98.2 (773)	97.7 (460)		92.6 (129)	87.0 (78)	
Yes	1.8 (11)	2.3 (6)		7.4 (12)	13.0 (13)	
**Ever received STI screening**			0.006			0.603
No	60.6 (489)	51.9 (248)		35.8 (65)	40.8 (36)	
Yes	39.4 (292)	48.1 (216)		64.2 (76)	59.2 (54)	
**Ever counselled about STIS**			0.902			0.221
No	25.9 (201)	25.5 (127)		44.2 (61)	52.1 (46)	
Yes	74.1 (568)	74.5 (333)		55.8 (77)	47.9 (44)	
**Number of partners last 12 months**			0.001			<0.001
<2	70.4 (570)	77.4 (364)		66.7 (97)	91.6 (78)	
≥2	29.6 (214)	22.6 (102)		33.3 (44)	8.4 (13)	
**Anal sex last 12 months**			0.326			0.057
No	36.6 (289)	41.1 (185)		39.9 (68)	55.7 (47)	
Yes	63.4 (492)	58.9 (276)		60.1 (73)	44.3 (39)	
**Condom use at last sex**				0.375		0.014
Yes	19.2 (152)	20.3 (98)		25.3 (29)	13.5 (14)	
No (regular partner)	75.5 (594)	76.4 (347)		64.2 (83)	83.2 (61)	
No (casual partner)	5.4 (35)	3.3 (16)		10.8 (11)	3.3 (3)	
**Depression score**			0.413			0.489
Score <10	80.9 (649)	82.9 (384)		66.8 (94)	71.0 (65)	
Score ≥10	19.1 (135)	17.1 (82)		33.2 (47)	29.0 (26)	
**Audit C score**			0.341			0.232
Non drinker	6.3 (46)	6.6 (30)		14.1 (20)	12.9 (11)	
Non risky drinker	56.9 (413)	52.1 (231)		55.6 (68)	48.8 (42)	
Risky drinker	36.7 (313)	41.3 (192)		30.3 (49)	38.3 (37)	

^a^ The study sample includes respondents who ever had sexual intercourse and have biological test results.

^b^ The excluded sample includes respondents who ever had sexual intercourse with no biological test results.

^**c**^ All percentages are weighted to account for complex sampling design and post stratification weights.

### Prevalence of diagnosed and self-reported STIs

**Fig 2:**
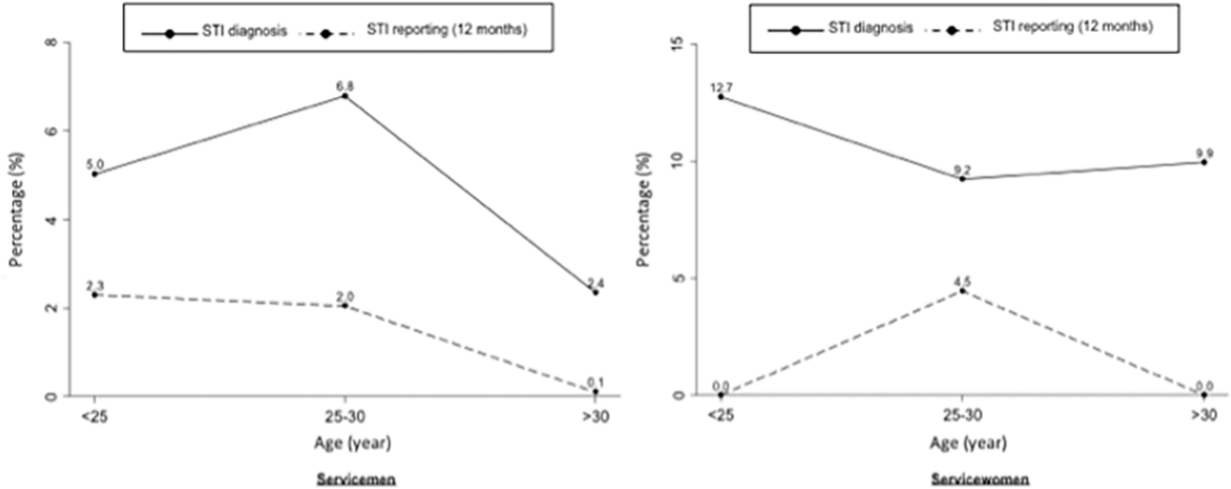
Prevalence of STI diagnosis and STI reporting (12 past months) according to gender and age class, COSEMIL survey.

The prevalence of diagnosed STIs was estimated at 4.7% [3.8–5.9], but varied substantially by sex (Table 3) and age (Fig 2). Female respondents were more than twice as likely to be tested positive than male respondents (10.4% *versus* 4.1%, p = 0.007). Results from the Heckman probit selection model accounting for sample selection (participants who did not have a biological test), indicates a “corrected” male STI prevalence of 4.5% [3.3–5.7], with a ρ coefficient that did not differ from 0 (ρ = 0.7; p = 0.23), suggesting no selection bias. Among women, the Heckman prevalence rate was 11.3% [6.9–15.7] with a ρ coefficient significantly different from 0 (ρ = -0.8; p = 0.04), indicative of selection bias.

**Table 3:** Prevalence of STIs among French service members, according to gender, and according to the origin of information (reported *versus* biologically confirmed (diagnostic STIs)), COSEMIL survey.

	Total	Men	Women	
	% ^b^ [95%CI] (N)	% ^b^ [95%CI] (N)	% ^b^ [95%CI] (N)	p
Reported STIs^a^ in the last 12 months	1.1 [0.6,2.3] (13/922)	1.1 [0.5,2.3] (12/781)	1.8 [0.8,4.1] (1/141)	0.282
Diagnostic STIs at the time of the survey	4.7 [3.8,5.9] (42/925)	4.1 [3.0,5.5] (31/784)	10.4 [6.2,16.8] (11/141)	**0.007**

^a^ Reported STIs included Syphilis, *Chlamydia trachomatis*, *Neisseria gonorrhea*, *Mycoplasma genitalium*

^**b**^ All percentages are weighted to account for complex sampling design and post stratification weights.

*Chlamydia* infections were the most common STIs detected with biological tests for men and women. Twenty-nine individuals were tested positive for *Chlamydia trachomatis* (3.4% 95%CI [2.4–5.0]), yielding a prevalence rate of *7*.1% (95% CI [3.2–11.0]) for women and 3.0% (95% CI [1.7–4.4]) for men. Applying the Heckman model to these rates, the corrected *Chlamydia trachomatis* prevalence rate was 8.2% for women and 3.0% for men. Twelve individuals were positive for *Mycoplasma genitalium*, yielding a prevalence rate of 1.4% (95% CI [1.0–1.8]), with 3.3% (95% CI [0.5–6.1]) for women and 1.2% (95% CI [0.8–1.5]) for men. Finally, one test was positive for *Neisseria gonorrhea* and one for syphilis. There were no cases of HIV or active hepatitis B.

Self- reported STIs in the last 12 months was evaluated at 1.1% [0.6–2.3], a rate that was four times lower (p<0.001) than diagnosed STI (Table 3). *Chlamydia* infections were the most commonly reported STIs. Information collected from the last *reported* case of STI for men indicated that most infections were contracted in Mainland France (n = 10) and a majority of infections were transmitted through contact with a casual partner (n = 6). An equal number of men sought treatment with a military physician or with a civilian physician. Only one woman reported an STI in the past 12 months, which was acquired with a regular partner in Mainland France.

While diagnosed STI rates significantly differed between men and women, the self-reported STI rates did not (1.1% *versus* 1.8%). However, the variation according to age was also observed (Fig 2). The sex difference in the discrepancy between reported and diagnosed STI estimates was substantial: female diagnostic cases of STIs relative to self-reported case were 5 times higher while there were four times higher for males (Table 3).

### Factors associated with diagnosed and self-reported STI

Associations between sociodemographic characteristics, sexual lifestyle and diagnosed STIs are reported in Table 4 for men and Table 5 for women. Respondents who were less educated, born overseas or in a foreign country, were in financially “tight” situations, had a lower military rank and were enrolled in the Army were more likely to have a prevalent case of diagnostic STI, although only educational attainment and place of birth remained significant in multivariate analysis for men (multivariate analysis was not performed for women due to small sample size). Recent deployment was not associated with STI diagnosis (results not shown). STI diagnosis was also related to religious practice. Few sexual lifestyle indicators were related to male and female STI diagnosis in bivariate analysis. Notably, number of partners in the last 12 months and use of condom at last intercourse were not related to STI diagnostic while cohabitation status for both sexes and anal sex in the last 12 months for men were associated in bivariate analysis. Only anal sex remained significant in multivariate analysis for males. STI counseling also tended to be associated with lower frequency of STI diagnosis while previous STI screening had no effect. Finally, men who reported depressive symptoms (score of 10 or + on CESD-10 scale) had lower odds of STI diagnostic while women who reported such symptoms were more likely to have a prevalent case of STI. Hazardous or harmful consumption of alcohol was not related to STI acquisition for both sexes.

**Table 4:** Factors associated with biologically confirmed STIs (diagnosed STIs)- results of univariate logistic regression among French servicewomen (n = 141) and servicemen (n = 784), COSEMIL survey.

		Women		Men	
		% ^a^ (N)	p-value		% ^a^ (N)	p-value
	**Total**	**10.4 (11/141)**		**4.1 (31/784)**	
**Age (years)**	<25	12.7 (5/40)	0.762	5.0 (9/154)	0.085
	25–30	9.2 (3/43)		6.8 (14/202)	
	>30	9.9 (3/58)		2.3 (8/428)	
**Cohabitation with current partner**	Everyday	8.6 (3/67)	0.156	2.4 (11/469)	**0.007**
	Not every day	19.0 (6/43)		7.0 (11/184)	
	No current partner	3.6 (2/30)		4.7 (8/130)	
**Level of education**	Less than High school	18.3 (5/36)	**0.017**	6.9 (21/313)	**<0.001**
	High school graduation	5.3 (4/77)		2.5 (8/300)	
	>High school	13.8 (2/27)		1.9 (2/169)	
**Financial situation**	No problem	8.6 (5/74)	0.392	2.4 (9/352)	**0.01**
	It’s difficult/It’s just enough	12.4 (6/66)		5.3 (22/430)	
**Place of birth**	Overseas France/Foreign country	9.9 (1/22)	0.941	10.0 (9/114)	**0.049**
	Mainland France	10.5 (10/119)		3.0 (22/670)	
**Children**	No	13.3 (10/86)	0.12	5.0 (18/347)	0.232
	Yes	3.1 (1/55)		3.3 (13/436)	
**Importance of religion**	Important/very important	29.3 (6/33)	**<0.001**	7.7 (11/149)	**p<0.001**
	Not very important/ not important	3.8 (2/67)		1.2 (7/409)	
	No religion	9.3 (3/41)		6.0 (13/218)	
**Military branch**	Army	5.7 (3/46)	**0.052**	6.0 (22/351)	**0.001**
	Air force	11.9 (3/55)		1.6 (5/238)	
	Navy	16.8 (5/40)		1.0 (4/195)	
**Army rank**	Enlisted personnel	11.7 (7/67)	0.581	6.0 (21/336)	**p<0.001**
	Officer/Noncommissioned Officer	9.4 (4/74)		2.7 (10/448)	
**At least one mission abroad**	No	14.3 (7/68)	**0.024**	5.0 (12/220)	0.138
	Yes	6.4 (4/73)		3.8 (19/564)	
**Number of years in the military**	1–10	10.4 (8/85)	0.994	5.9 (21/323)	**0.043**
	>10	10.3 (3/56)		2.7 (10/459)	
**Number of sex partners (12 months)**	<2	8.2 (5/97)	0.07	2.6 (16/570)	0.069
	≥2	14.7 (6/44)		7.7 (15/214)	
**Oral sex (12 months)**	No	15.6 (2/14)	0.346	4.1 (4/104)	0.993
	Yes	9.9 (9/127)		4.1 (27/680)	
**Anal sex (12 months)**	No	12.2 (10/107)	0.138	2.2 (14/526)	**0.032**
	Yes	5.8 (1/34)		7.4 (16/256)	
**Ever had commercial sex**	No			4.8 (18/459)	0.577
	Yes			3.1 (13/325)	
**Ever received STI screening**	No	5.1 (3/65)	0.121	4.0 (18/489)	0.841
	Yes	13.3 (8/76)		4.2 (13/292)	
**Ever counselled about STIS**	No	15.2 (6/61)	0.062	6.9 (13/201)	**0.018**
	Yes	6.7 (5/77)		3.2 (18/568)	
**Condom use at last sex**	No	11.1 (7/94)	0.644	4.0 (24/629)	0.858
	Yes	13.2 (4/29)		4.5 (7/152)	
**Depression**	No	12.3 (9/94)	0.258	4.8 (28/649)	0.062
	Yes	6.4 (2/47)		1.0 (3/135)	
**Audit C**	Non drinkers	- (0/20)	0.11	1.4 (2/46)	0.297
	Non risky drinkers	14.0 (8/68)		3.9 (13/413)	
	Risky drinkers	9.3 (3/49)		4.8 (15/313)	

^**a**^ All percentages are weighted to account for complex sampling design and post stratification weights.

**Table 5:** Factors associated with biologically confirmed STIs (diagnosed STIs)- results of multivariate logistic regression (n = 753), COSEMIL survey.

		AOR^a^	[CI95%]	p
**Age (years)**	<25 years	1.29	[0.29,5.82]	0.77
	25–30 years	1.4	[0.52,3.79]	
	> 30 years	1		
**Cohabitation with current partner**	Everyday	1		0.11
	Not every day	1.84	[0.32,10.75]	
	No current partner	2.91	[1.10,7.71]	
**Educational level**	Less than High school	3.12	[1.23,7.91]	**0.06**
	High School graduation	1		
	> High school	1.12	[0.19,6.73]	
**Financial situation**	No problem	1		0.2
	It’s difficult / It’s just enough	1.34	[0.84,2.16]	
**Place of birth**	Mainland France	1		**0.002**
	Overseas France / Foreign country	2.46	[1.51,4.01]	
**Religiosity**	Important / very important	4.45	[1.69,11.74]	**0.008**
	Not really / not important at all	1		
	No religion	4.64	[1.99,10.84]	
**Military branch**	Army	1		0.17
	Air force	0.37	[0.13,1.07]	
	Navy	0.36	[0.08,1.65]	
**Military rank**	Enlisted personnel	0.55	[0.10,2.93]	0.5
	Noncommissioned Officer	1		
	Officer	0.34	[0.02,6.78]	
**Number of sex partners (12 months)**	<2	1		0.58
	≥2	1.74	[0.21,14.47]	
**Had anal sex in the last 12 months**	No	1		**0.04**
	Yes	1.91	[1.04,3.53]	
**Ever had commercial sex**	No	1		0.59
	Yes	0.64	[0.11,3.77]	
**Ever counseled about STIS**	No	1.94	[0.78,4.84]	0.14
	Yes	1		
**Condom use at last sex**	No	1		0.69
	Yes	0.61	[0.04,8.88]	
**Depression**	No	1		**0.04**
** **	Yes	0.16	[0.03,0.90]	

^**a**^ Analysis is weighted to account for complex sampling design and post stratification weights.

The small number of cases of reported STIs in the last 12 months (12 men and 1 women) precludes any analysis of associations for women and precludes multivariate analysis for men. Thus, we only present bivariate associations for men in Table 4. Results indicate significant differences in the determinants of reported *versus* diagnostic STIs. In particular, age and sexual orientation were associated with reported STIs, but not with diagnostic STIs. Conversely, cohabitation status, STI counseling and depression were associated with STI diagnosis but not with reported STIs.

## Discussion

Prevalence of STI diagnosis in the French armed forces was estimated at 4.7% [3.8, 5.9], 4 times higher than the 12 months self-reported STI rate evaluated at 1.1% [0.6, 2.3]. *Chlamydia trachomatis* was the most common infection, with a prevalence of 7.1% [3.2, 11.0] for women and 3.0% [1.7, 4.4] for men. As reported in other military settings, STI rates in our military population were substantially higher than in the general population, especially for women, where the prevalence of *Chlamydia trachomatis* was almost three times higher than the 1.6% [1.0–2.5] diagnosed among French women aged 18 to 44 years [19,39,40]. For men, *Chlamydia trachomatis* was 2 times higher in our military population as compared to the 1.4% [0.8–2.6] prevalence among French men aged 18 to 44 years [39]. These higher estimates may partly reflect the younger distribution in the military as compared to the general population, although the highest prevalence of diagnostic cases of Chlamydia estimated among women 18–24 years in the general French population was 3.6% which is substantially lower than the 12.7% observed among servicewomen of the same age group. Likewise, the 6.8% prevalence rate of Chlamydia prevalence among servicemen aged 25 to 29 was substantially higher than the 2.7% observed among men of the same age in the general population (who had the highest prevalence rate of all age groups) [41].

The present study identifies a number of risk factors for STIs that fall into four categories (demographics, sexual behaviors, socio-cultural factors and health-related factors), differ between measures (STI diagnosis *versus* self-reports) [20,42] and differ by sex.

The highest burden of STIs in our study was observed among young servicewomen, with a prevalence reaching 12.7% among women under the age of 25 years. Such sex and age patterns have previously been reported among US military personnel [27,40,43] leading the U.S. Preventive Services Task Force to recommend yearly screening of STIs for all women less than 25 years [32,44]. Ranked among the 10 most cost-effective prevention strategies by the National Commission on Prevention Priorities [45], such annual screening currently reaches up to 85% of the target US military population in 2014, with an associated 15% projected decline in *Chlamydia* rates between 2011 and 2014 [46]. The French military health service has yet to introduce such a gender approach to STI screening and prevention, which currently focuses on male sexual risks related to commercial sex or multi-partnerships during deployment. In our study 76% of servicewomen partner with military men, which increases the risk of transmission within the military community. Addressing military women’s health care needs may thus serve as a general strategy to reduce STI rates among the military.

Sexual risk behaviors were commonly reported by servicemen who were 41% to indicate ever having had commercial sex and 29.6% to report multi-partnerships in the last 12 months. In addition, 36.7% of servicemen were classified as “risky drinkers” (a proportion that was substantially higher than the 20% reported among men 18–59 participating in the French workforce [47]), yet these behaviors were not predictive of STI diagnosis in our study. This is in contrast to prior studies linking binge drinking to sexual risk taking behaviors including inconsistent use of condoms, in military populations [11,21,48]. The French military health program, which offers education and unlimited free access to condoms during deployment abroad may contribute to better protection during male risky sexual encounters such as commercial sex or multi-partnerships, particularly during deployment. The low percentage of men who indicated not using a condom at last sex with a casual partner; the absence of effect of recent deployment and the lower odds of STI diagnosis among men who received STI counseling may reflect such an influence. Anal sex is also a commonly described risk factor for male STI acquisition, but usually addressed in the context of men who have sex with men. In our study, anal sex in the last 12 months was related to both STI diagnosis and self-reported STI for men, mostly in the context of heterosexual relations: all STI diagnosis were to men who only reported heterosexual intercourse in the last 12 months. In the absence of an impact evaluation of STI prevention programs in the French military, the influence of the French military health program on STI rates is unknown, but future research assessing the impact of a revised program based on COSEMIL findings should fill in this gap.

Current military preventive strategies seem unable to curtail social and regional disparities in STI acquisition, as we found a higher burden of STI diagnosis among the least educated service-members and those who were born in potential high STI endemic environments. Higher STI rates among less educated individuals were also found in the general population in France [41] as well as in military settings in other countries. In such studies, authors also report on other social disparities by racial/ethnicity and military rank [26,27,40,49,50]. Race/ethnicity was not assessed in the COSEMIL survey (the French ethical review board prohibits data collection on race and ethnicity) and we found no effect of military rank after adjusting for level of education, probably signaling lack of educational variation by rank. Finally, we report differences in rates of STI by religiosity, with greater risk in non-religious men and women and highly religious men. Lack of religiosity has previously been described as a risk factor for STI acquisition in the US [51], but greater religiosity has not, suggesting religiosity is indicative of socio-cultural practices that vary across societies and by gender.

A number of studies conducted in the military have described higher rates of STIs with respect to mental health issues (depression and post-traumatic stress) [52–55]. In the present study, we found an inverse association with depressive symptoms among men. Such gender differences in the association between STI and depressive symptoms have not been reported previously and warrant further investigation.

Our study has several limitations. While prevalence of STIs was higher among women, the small sample of female recruits, despite their oversampling by 5 to 1, constrained our analysis of risk factors for STIs among women. Another limit relates to refusal rates, typical in population based studies (the acceptance rate for the Natsal study was 71% and for the CSF survey in France was 76% [39]) and the high percentage of unusable samples, resulting in potential selection bias and decreased statistical power. Using Heckman’s selection approach, we attempted to account for selection bias and provided corrected estimates of 4.5% among men and 11.3% among women. While this approach improves our initial estimates, we acknowledge that selection bias may still affect the validity of our results.

Despite these limitations, we believe the present study provides guidance to adapt military health services to address the current ecology of STIs in the Armed Forces, as well as important insights on the potential biases of using self-reports as the sole measure of STIs in population based studies.

## Conclusion

Results of this study underline the need to use STI biomarkers in population based studies, given the differential and substantial underreporting of STIs leading to biased results. The study also highlights the need for programmatic adaptation of the French military STI task force to address gender inequalities in STI infections, by developing women’s health services in the French military. Addressing military women’s health care needs not only benefits women themselves but may also serve as a general strategy to reduce STI rates as most women in the military partner with military men increasing the risk of internal transmission.

## Supporting information

S1 Appendix Detailed biological methods used for pathogens diagnostics, COSEMIL survey. (DOCX)
