# Supplementary material for: Prevalence and risk factors of sexually transmitted infections among French service members
Source: PLoS One. 2018 Apr 2;13(4):e0195158. doi: 10.1371/journal.pone.0195158 (PMC5880385; doi:10.1371/journal.pone.0195158)
Supplement: S1 Appendix — (DOCX) [file pone.0195158.s001.docx]

**S1 Appendix : Detailed biological methods used for pathogens diagnostics, COSEMIL survey**

| Biological test | Technique used |
| --- | --- |
| HIV serology | ELISA serology combining Ac-antiHIV1/2 and AgP24 detection: HIV Combi Elecsys, Cobas, Roche |
| HBV serology | Anti-HBc Elecsys, AntiHBs Elecsys, AgHBs Elecsys |
| HCV serology | Anti-HCV Elecsys |
| Syphilis serology | Cobas TPLA Elecsys, Roche and VDRL Check, Alldiag |
| *Chlamydia trachomatis* PCR | Combined nucleic acid amplification test with real-time PCR method:  Dx CT/NG/MG assay with Dx real-Time System, Biorad, Marnes-la-Coquette, France |
| *Neisseria gonorrhea* PCR |  |
| *Mycoplasma genitalium* PCR |  |
